# Deep learning-based fetoscopic mosaicking for field-of-view expansion

**DOI:** 10.1007/s11548-020-02242-8

**Published:** 2020-08-17

**Authors:** Sophia Bano, Francisco Vasconcelos, Marcel Tella-Amo, George Dwyer, Caspar Gruijthuijsen, Emmanuel Vander Poorten, Tom Vercauteren, Sebastien Ourselin, Jan Deprest, Danail Stoyanov

**Affiliations:** 1 Wellcome/EPSRC Centre for Interventional and Surgical Sciences (WEISS) and Department of Computer Science, University College London, London, UK; 2 Department of Mechanical Engineering, KU Leuven University, Leuven, Belgium; 3 School of Biomedical Engineering and Imaging Sciences, King’s College London, London, UK; 4 Department of Development and Regeneration, University Hospital Leuven, Leuven, Belgium

**Keywords:** Deep learning, Surgical vision, Twin-to-twin transfusion syndrome (TTTS), Fetoscopy, Sequential mosaicking

## Abstract

**Purpose:**

Fetoscopic laser photocoagulation is a minimally invasive surgical procedure used to treat twin-to-twin transfusion syndrome (TTTS), which involves localization and ablation of abnormal vascular connections on the placenta to regulate the blood flow in both fetuses. This procedure is particularly challenging due to the limited field of view, poor visibility, occasional bleeding, and poor image quality. Fetoscopic mosaicking can help in creating an image with the expanded field of view which could facilitate the clinicians during the TTTS procedure.

**Methods:**

We propose a deep learning-based mosaicking framework for diverse fetoscopic videos captured from different settings such as simulation, phantoms, ex vivo, and in vivo environments. The proposed mosaicking framework extends an existing deep image homography model to handle video data by introducing the controlled data generation and consistent homography estimation modules. Training is performed on a small subset of fetoscopic images which are independent of the testing videos.

**Results:**

We perform both quantitative and qualitative evaluations on 5 diverse fetoscopic videos (2400 frames) that captured different environments. To demonstrate the robustness of the proposed framework, a comparison is performed with the existing feature-based and deep image homography methods.

**Conclusion:**

The proposed mosaicking framework outperformed existing methods and generated meaningful mosaic, while reducing the accumulated drift, even in the presence of visual challenges such as specular highlights, reflection, texture paucity, and low video resolution.

**Electronic supplementary material:**

The online version of this article (10.1007/s11548-020-02242-8) contains supplementary material, which is available to authorized users.

## Introduction

**Fig. 1:**
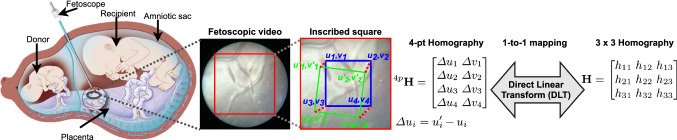
Pictorial illustration of the fetoscopic laser photocoagulation for the treatment of TTTS and 1-to-1 mapping of 4-pt and $3\times 3$ homography parameterizations

Twin-to-twin transfusion syndrome (TTTS) is a rare condition during pregnancy that affects 10–15% of genetically identical twins sharing a monochorionic placenta [5]. It is caused by abnormal placental vascular anastomoses on the chorionic plate of the placenta resulting in uneven flow of blood between the fetuses. This condition puts both twins at risk and requires treatment before birth to increase their survival rate. Fetoscopic laser photocoagulation (Fig. 1), a minimally invasive procedure, is the most effective treatment for TTTS in which the surgeon uses a fetoscopic camera to inspect and identify abnormal vascular anastomoses on the placental chorionic plate, and uses a retractable laser ablation tool in the working channel of the scope to photocoagulate the vascular anastomoses to separate the blood circulation of each twin. Limited field of view (FoV) and maneuverability of the fetoscope, poor visibility [18] due to variability in light source, and unusual placenta position [11] may impede the procedure leading to increased procedural time and incomplete ablation of anastomoses resulting to persistent TTTS. Fetoscopic mosaicking can create an expanded FoV image of the placental surface, which may facilitate the surgeons in localizing vascular anastomoses during the procedure.

Mosaicking for the FoV expansion in fetoscopy has been explored using feature-based, intensity-based, and deep learning-based methods [2, 3, 10, 21, 22, 26, 27]. Reeff et al. [22] and Daga et al. [10] used the classical image feature-based matching method for creating mosaics from planar placenta images. Reeff et al. [22] experimented with the fetoscopic images of an ex vivo placenta submerged in water, while Daga et al. [10] used images of an ex vivo phantom placenta. A mosaic is generated by aligning the relative transformations between the pair of consecutive fetoscopic images with respect to a reference frame. A small error in the relative transformations can introduce large drift in the mosaic, where global consistency alignment techniques and use of electromagnetic tracker can help to minimize the drifting error [21, 26, 27]. Tella-Amo et al. [26, 27] assumed placenta to be planar and static and integrated the electromagnetic tracker with the fetoscopic in a synthetic and ex vivo setup to propose a mosaicking approach capable of handling the drifting error. However, current clinical regulations and limited form factor of the fetoscope hinder the use of such a tracker in intraoperative settings. Peter et al. [21] proposed a direct pixel-wise alignment of gradient orientations for creating a mosaic from a single in vivo fetoscopic video. Gaisser et al. [15] detected stable regions on veins of the placenta using a region-based convolutional neural network and then used these detected regions as features for placental image registration in an underwater phantom setting [15]. Bano et al. [3] proposed a deep learning approach for placental vessel segmentation and registration for mosaicking and showed that vessels can act as unique landmarks for creating mosaics with minimum drifting error. Mosaicking from fetoscopic videos particularly remains challenging due to fetoscopic device limitations (monocular low-resolution fetoscopic camera with FoV limitation), occlusion by the fetus, ablation tool presence and occasional bleeding, non-planar views, turbid amniotic fluid, specular highlights and reflection due to variation in the light source, distortion due to light refraction [9], and texture paucity. Automatic selection of occlusion-free fetoscopic video segments [4] can help in identifying relevant segments for mosaicking. We showed in [2] that deep learning-based image alignment helps in reducing the accumulated drift, even in the presence of visual challenges such as specular highlights, reflection, texture paucity, and low video resolution.

Supervised deep learning-based techniques estimate the correspondences between pair of disparate natural scene images [13, 23, 25] by using benchmark datasets of disparate natural scene images with known ground-truth correspondence for training. However, ground-truth correspondences are unknown in fetoscopic videos. Moreover, [13] and [25] used pair of high-resolution natural scene images which are sharp and rich in both texture and color contrast, contrary to fetoscopic videos which are of low resolution, lack both texture and color contrast since the in vivo scene is monotonic in color, and are unsharp because of the introduced averaging to compensate for the honeycomb effect of the fiber bundle scope. As a result, hand-crafted feature-based methods perform poorly on the fetoscopic videos. Shen et al. [23] and Srivastava et al. [25] used pretrained deep learning features as backbone for learning the correspondences between natural images. However, in the case of fetoscopic videos, due to poor texture and contrast, feature maps computed from pretrained networks may not capture distinct features for robust correspondence estimation since these models are pretrained on natural images (like ImageNet) which does not capture the fetoscopic data distribution. Moreover, none of the approaches [13, 23, 25] extended beyond pair of images matching to expand the field of view from videos.

Self-supervised deep learning-based solutions can overcome some of the challenges associated with fetoscopic mosaicking. Image homography estimation methods have been proposed [12, 20] that use pairs of image patches extracted from a single image to estimate the homography between them. In practice, a full mosaic is generated by computing sequential homographies between adjacent frames in an image sequence, where fetoscopic video poses additional challenges due to artifacts and occlusions, thus affecting the stitching problem. However, such challenges can be tackled by estimating the homographies between a pair of consecutive frames by extracting random pair of patches each time and estimating the most consistent homography. In this paper, we adopt this approach and propose a framework for mosaicking from fetoscopic videos captured from various fetoscopes and in various experimental settings. We adapt the deep image homography (DIH) [12] estimation method for training by assuming that the transformation between two adjacent frames contains a rotation and translation component only. We propose a controlled data generation approach that uses a small set of fetoscopic images of varying quality and appearance, for training. We then perform outlier rejection to find the consistent homography estimate between multiple pair of patches extracted at random from two consecutive frames. Controlled data generation and outlier rejection combine to minimize the drift without the use of any external sensors. We perform comparison of the proposed fetoscopic video mosaicking (FVM) framework with existing methods using 5 diverse datasets. This paper is an extended version of the work presented at the MICCAI 2019 conference [2] and provides a broader insight into the fetoscopic mosaicking research, comprehensive analysis of both qualitative and quantitative results and comparison with the existing methods.

## Problem formulation

A homography (rigid) or projective transformation, represented by a $3\times 3$ matrix ${\mathbf {H}}$, is a nonlinear transformation that maps image points $\mathbf {x} \rightarrow \mathbf {x}'$ between two camera views under the planar scene assumption:

1 $$\begin{aligned} \begin{bmatrix} {u}'\\ {v}'\\ 1 \end{bmatrix} \propto \begin{bmatrix} h_{11} &{} \quad h_{12} &{} \quad h_{13} \\ h_{21} &{} \quad h_{22} &{} \quad h_{23} \\ h_{31} &{} \quad h_{32} &{} \quad h_{33} \end{bmatrix} \begin{bmatrix} u\\ v\\ 1 \end{bmatrix}, \end{aligned}$$

where (*u*, *v*) is a 2D point that is mapped to $(u',v')$ with the homography ${\mathbf {H}}$ and ${\mathbf {H}}$ is defined up to a scale; hence, it has eight degrees of freedom.

The problem of generating a mosaic from an image sequence is to find the pairwise homographies between frames F$_k$ and F$_{k+l}$ (where *k* and *l* are not necessarily consecutive frames) followed by computing the relative homographies with respect to a fixed reference frame, also termed as the mosaic plane. The relative homography is represented by left-hand matrix multiplication of pairwise homographies:

2 $$\begin{aligned} {\mathbf {H}}_{k+l}^{k}= \prod _{i = k}^{k+l-1}{\mathbf {H}}_{i+1}^{i}. \end{aligned}$$

This assumes a piecewise planarity of the placental surface observed by the fetoscope, and while not generally true, it is sufficient for local patches of the placenta. Note that a rigid transformation model is considered since the placental surface does not deform over time. Moreover, there is no perceptible placental vessel expansion/contraction due to the breathing of the patient.

**Fig. 2:**
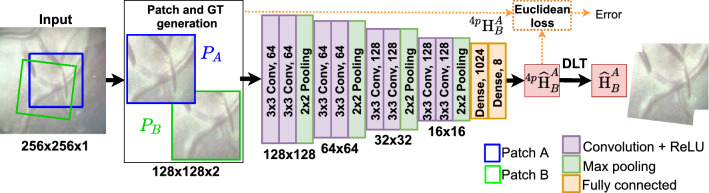
DIH regression network with controlled data generation for training

## Deep image homography

Deep image homography (DIH) model [12] uses a convolutional neural network to estimate the relative homography between pairs of image patches extracted from a single image by learning to estimate the four-point homography.

### Four-point homography estimation

The rotation and shear components in the 3 $\times$ 3 parameterization ${\mathbf {H}}$ have smaller magnitude compared to the translation; as a result, their effect on the loss function during training is small. Therefore, DIH model [12] uses the 4-point homography parameterization $^{4p}{\mathbf {H}}$ [1], instead of the 3$\times$3 parameterization ${\mathbf {H}}$ (Eq. 1) for the estimation. Let $(u_i,v_i)$, where $i =1,2,3,4$, denote the four corners of an image $P_A$ and $(u_i',v_i')$ denote the four corners in an overlapping image $P_B$. Then, $\Delta u_i = u_i' - u_i$ and $\Delta v_i = v_i' - v_i$ give the displacement of the *i*th corner point, and the 4-point homography parameterization $^{4p}{\mathbf {H}}$ is given by:

3 $$\begin{aligned} ^{4p}{\mathbf {H}} = \begin{bmatrix} \Delta u_1 &{} \quad \Delta u_2 &{} \quad \Delta u_3 &{} \quad \Delta u_4\\ \Delta v_1 &{} \quad \Delta v_2 &{} \quad \Delta v_3 &{} \quad \Delta v_4 \end{bmatrix}^{T}. \end{aligned}$$

A one-to-one mapping exists between the 4-point $^{4p}{\mathbf {H}}$ and 3 $\times$ 3 ${\mathbf {H}}$ homography parameterizations. With the $(u_i,v_i)$ and $(u_i',v_i')$ known in Eq. 1, ${\mathbf {H}}$ can be computed by applying direct linear transform [16] (Fig. 1).

DIH is a VGG-like [24] network (Fig. 2) which is used for regressing the displacement between the four corner points. The network consists of 8 convolutional layers and 2 fully connected layers. The input of the network is ${P}_{A}$ and ${P}_{B}$ extracted at random from a single image, and output is their relative homography $^{4p}\widehat{{\mathbf {H}}}_{B}^{A}$. For the gradient back-propagation during the training process (represented by dotted line in Fig. 2), the network uses the Euclidean loss

4 $$\begin{aligned} L_2 = \frac{1}{2}\left\| ^{4p}{\mathbf {H}} - ^{4p}\widehat{{\mathbf {H}}} \right\| ^2, \end{aligned}$$

where $^{4p}{\mathbf {H}}$ and $^{4p}\widehat{{\mathbf {H}}}$ are the ground-truth (GT) and predicted 4-point homographies. Note that [12] used the MS-COCO dataset for training, where pair of patches were extracted from a single static real-world image, free of artifacts (e.g., specular highlights, amniotic fluid particles) that appear in sequential data.

### Limitation of deep image homography

For the DIH model, the training data are generated by randomly selecting an image patch ${P}_{A}$ of size $128 \times 128$ from a grayscale image and randomly perturbing all its four corners by a maximum of 32 pixels to obtain ${P}_{B}$ and the relative GT $^{4p}{\mathbf {H}}_{B}^{A}$. It is observed through experimentation that data generation by performing random perturbation (as proposed in [12]) results in scenarios that are difficult for the network to learn, hence resulting in higher error. In the case of mosaicking, where homography between frames F$_k$ and F$_{k+l}$ is computed by accumulating the intermediate pairwise homographies (Eq. 2), even a small error in pairwise homography will accumulate over time resulting in increasing drift. Therefore, this data generation model cannot be used as it is for creating mosaics from sequential data.

**Fig. 3:**
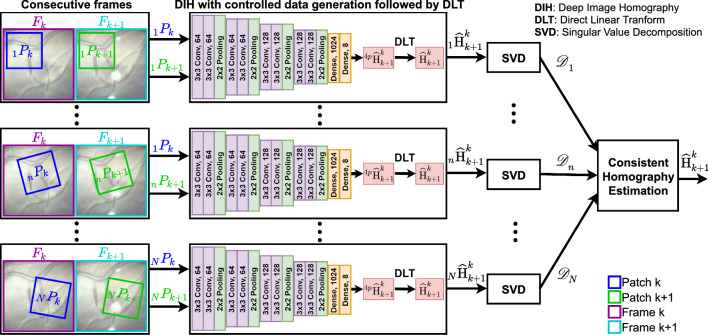
Overview of the proposed FVM framework

## Fetoscopic video mosaicking

An overview of the proposed fetoscopic video mosaicking (FVM) is shown in Fig. 3, which can be divided into two stages, (1) data generation for regression network training (Sect. 4.1) and (2) consistent homography estimation (Sect. 4.2). To overcome the limitation of DIH, we propose controlled data generation in which DIH is trained on pairs of patches generated by introducing translation and rotation transformations only on a single image. During testing, we apply the median filter to decomposed homography estimations from different patches of the same pair of consecutive frames to get a robust estimate of the homography.

### Data generation for regression network training

In sequential data, pairwise homography between two adjacent frames F$_k$ and F$_{k+1}$ is related by affine transformations including rotation, translation, scale, and shear, where the scale can be considered as constant since fetoscopy is performed at a fixed distance from the placental surface. Moreover, the motion of the fetoscope is physically constrained by the incision point (remote center of motion), making shear component extremely small compared to the translation and rotation components. Therefore, scale and shear components can be neglected. We assume that F$_k$ and F$_{k+1}$ are related by the translation and rotation components only. This assumption helps in minimizing the error in relative homography between frames.

For controlled data generation, given a grayscale image *I* of size $256\times 256$, an image patch $P_A$ of size $128\times 128$ is extracted at a random location with corner points given by $(u_i, v_i)$, where $i = 1, 2, 3, 4$. The four corners for $P_B$ are then computed by applying a rotation by $\beta$ and translation by $(d_x,d_y)$ to $(u_i, v_i)$:

5 $$\begin{aligned} \begin{bmatrix} u_i'\\ v_i' \end{bmatrix} = \begin{bmatrix} \mathrm{cos}\beta &{} \quad \mathrm{sin} \beta \\ -\mathrm{sin} \beta &{} \quad \mathrm{cos} \beta \end{bmatrix} \begin{bmatrix} u_i\\ v_i \end{bmatrix} +\begin{bmatrix} d_x\\ d_y \end{bmatrix}, \end{aligned}$$

where $\beta$, $d_x$ and $d_y$ are empirically selected. $^{4p}{\mathbf {H}}_B^A$ is then obtained using Eq. 3. $P_A$, $P_B$, and $^{4p}{\mathbf {H}}_B^A$ form the input of the regression network. During training (Fig. 2), the relative homography is learned between patches that are extracted from a single image following the controlled data generation process. During testing where the patches are extracted from the consecutive frames, the homography estimate may not always be accurate due to texture paucity and poor contrast in fetoscopy.

### Consistent homography estimation

Outlier rejection is performed during testing by applying median filtering to the homographies estimates obtained from random patches of the same pair of frames (Fig. 3). To obtain a robust estimate of the homography, we first need to decompose $\mathbf {\widehat{H}}$. We apply singular value decomposition [19] which decomposes ${\mathbf {H}}$ into a rotation, a non-uniform scale, and another rotation, given by:

6 $$\begin{aligned} \begin{bmatrix} \widehat{h}_{11} &{} \quad \widehat{h}_{12} \\ \widehat{h}_{21} &{} \quad \widehat{h}_{22} \end{bmatrix} = \begin{bmatrix} \mathrm{cos}\widehat{\theta } &{} \quad \mathrm{sin} \widehat{\theta } \\ -\mathrm{sin} \widehat{\theta } &{} \quad \mathrm{cos} \widehat{\theta } \end{bmatrix} \begin{bmatrix} \widehat{s}_g &{} \quad 0 \\ 0 &{} \quad \widehat{s}_h \end{bmatrix} \begin{bmatrix} \mathrm{cos}\widehat{\gamma } &{} \quad \mathrm{sin} \widehat{\gamma } \\ -\mathrm{sin} \widehat{\gamma } &{} \quad \mathrm{cos} \widehat{\gamma } \end{bmatrix}, \nonumber \\ \end{aligned}$$

where $\widehat{\theta }, \widehat{\gamma }, \widehat{s}_g$ and $\widehat{s}_h$ are the unknowns. Since the left-hand side in Eq. 6 is known, we can solve the simultaneous equations for $\widehat{\theta }, \widehat{\gamma }, \widehat{s}_g$ and $\widehat{s}_h$ (for derivation refer to [7]). The translation components can be extracted directly from $\mathbf {\widehat{H}}$ as $\widehat{t}_x = \widehat{h}_{13}$ and $\widehat{t}_y= \widehat{h}_{23}$. For affine transformation, the homography parameters $\widehat{h}_{31} \simeq 0$ and $\widehat{h}_{32} \simeq 0$. In Eq. 6, the rotation matrices are orthogonal and the scale matrix is diagonal.

For a pair of consecutive frames F$_k$ and F$_{k+1}$, we compute the homography $_n\widehat{{\mathbf {H}}}_{k+1}^{k}$ for $N=99$ iterations such that at each iteration, a new random pair of patches $_n$P$_k$ and $_n$P$_{k+1}$ is used. This results in slightly varying estimations at some iterations due to varying visual quality across the sequence. For each iteration, we obtain the decomposed parameters where the rotation components can be represented as $(\widehat{\theta }_n)_{n=1}^{N}$ and $(\widehat{\gamma }_n)_{n=1}^{N}$. The variations in scale components are very small due to fixed scale assumption during the training process. But the variations in the rotation components are significant and useful for outlier rejection. Hence, we compute the median over all the iterations for $(\widehat{\theta }_n)_{n=1}^{N}$ to get its argument:

7 $$\begin{aligned} \widehat{\theta }_i = \underset{n}{\mathrm{arg}\,\,median }((\widehat{\theta }_i)_{n = 1}^{N}), \end{aligned}$$

which gives the most consistent value for $\theta$. The values of $\widehat{\theta }_i$, $\widehat{\gamma }_i$, $\widehat{s}_{xi}$, $\widehat{s}_{yi}$, $\widehat{t}_{xi}$ and $\widehat{t}_{yi}$ are then plugged into Eq. 6 to get the consistent $_i\widehat{{\mathbf {H}}}_{k+1}^{k}$.

## Experimental details

### Dataset

For the experimental analysis, we use five fetoscopic videos that captured phantom and real human placenta in ex vivo and in vivo environments. Our video data include 2 videos from the existing literature, namely synthetic (SYN)—a discontinuous version of this sequence was used in [26], and an ex vivo in water (EX) data reported from [14]. We also captured two videos using placenta phantom in in-house settings. The first phantom video, referred as PHN1, was captured using a rigid placenta model in air placed in a surgical trainer box [17]. The second phantom video, referred as PHN2, was captured using a TTTS phantom.^1^ PHN1 and PHN2 were captured with Storz rigid $30^{\circ }$ and $0^{\circ }$ scopes, respectively, having light source in one of their working channels. The fifth video sequence is from an in vivo TTTS procedure (INVI) that intraoperatively captured the human placenta. PHN1, PHN2, and INVI differ significantly from SYN and EX as they captured non-planar views with freehand motion, thus creating challenging scenarios for mosaicking methods.

**Table 1:** Main characteristics of the videos used for the experimental analysis

Video name	Imaging source	# Frames	Frame resolution (pixels)	Cropped frame resolution (pixels)	Camera view	Motion type
Synthetic (SYN) [26]	–	811	385 $\times$ 385	260 $\times$ 260	Planar	Circular
Ex vivo in water (EX) [14]	Stereo	404	250 $\times$ 250	250 $\times$ 250	Planar	Spiral
Phantom without fetus (PHN1)	Rigid $30^{\circ }$ scope	681	1280 $\times$ 960	834 $\times$ 834	Non-planar	Circular (freehand)
TTTS Phantom in water (PHN2)	Rigid scope	350	720 $\times$ 720	442 $\times$ 442	Non-planar with heavy occlusions	Exploratory (freehand)
In vivo TTTS procedure (INVI)	Rigid scope	150	470 $\times$ 470	312 $\times$ 312	Non-planar with heavy occlusions	Exploratory (freehand)

**Fig. 4:**
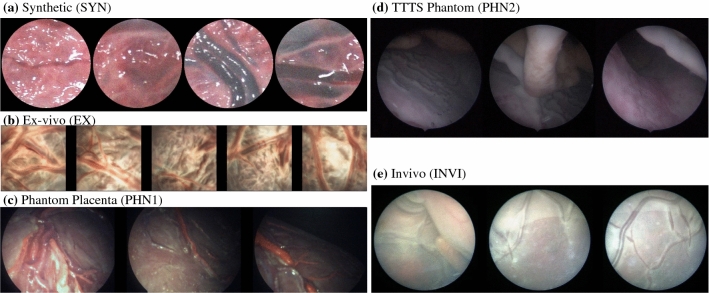
Representative frames from the five videos under analysis

Table 1 summarizes the main characteristics of the five test videos, and representative images from these videos are shown in Fig. 4. The visual quality, appearance, frame resolution, and imaging source vary across all five videos. These variations pose challenging scenarios for mosaicking methods. SYN and EX captured a planar view and followed a circular loop closure and spiral motion, respectively. EX was captured using a KUKA articulated arm robot and followed a pre-programmed fixed spiral trajectory [14]. PHN1 captured non-planar views that followed a freehand circular trajectory depicting a scenario with loop closure. PHN2 and INVI captured highly non-planar views containing heavy occlusions.

### Experimental setup

The recorded videos are converted to frames using the FFmpeg software.^2^ To extract a square frame from the circular (Fig. 4), in order to use it as the input for the proposed network, we detect the circular mask of the scope through pixel-based image threshold and morphology. A square cropped frame is then extracted such that it is an inscribed square within the circular mask (Table 1). Note that the resolution of frames varies as they were captured from different imaging sources. For training (Sect. 4.1), we extracted 600 frames at random from SYN, PHN1, PHN2, INVI, and another in-house ex vivo still images dataset. Note that the still image ex vivo dataset is not a video sequence; hence, it was only used during training as it does not satisfy the continuous video assumption. EX dataset (Table 1) was not used during training; hence, it is a completely unseen data used for testing the generalization of the proposed method to an unseen dataset. We converted the training images to grayscale and resized them to $256 \times 256$ resolution. We use Keras with Tensorflow backend for the implementation and train the regression network for 15 hours on a Tesla V100 (32GB) using a learning rate of $10^{-4}$ and ADAM optimizer. Pairs of patches with controlled data augmentation (Sect. 4.1) are generated at run time in each epoch by randomly selecting $\beta$ between $(-5^{\circ },+5^{\circ })$, and $d_x$ and $d_y$ between $(-16,16)$. The regression network is trained for 60,000 epochs with a batch size of 32. Same training settings are used for training the regression model without controlled data augmentation where each corner point of $P_A$ is perturbed at random between $(-16,16)$.

**Fig. 5:**
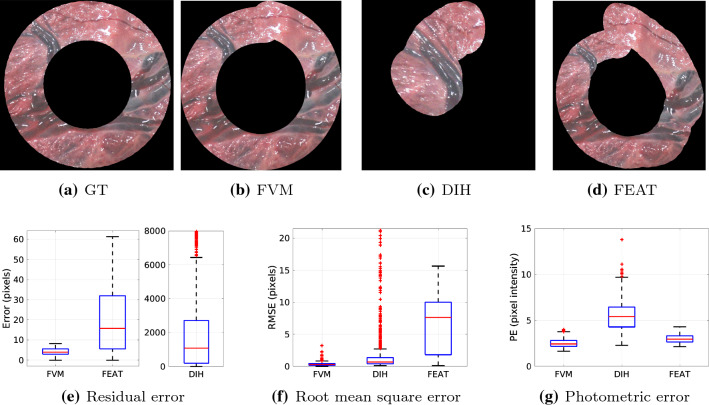
**a**–**d** Visualization of mosaics for one circular loop (360 frames) of the SYN sequence. **e**–**g** Quantitative comparison of FEAT, DIH and FVM

### Evaluation protocol

We perform comparison of the proposed FVM with a feature-based (FEAT) [8] and DIH [12] methods. FEAT extract speeded up robust features [6] from a pair of images and performs an exhaustive search for feature matching to estimate the homography. In fetoscopic videos, the GT pairwise homographies between consecutive frames are unknown. Hence, the accumulated error over time can mainly be observe through qualitative analysis. The GT homographies are only available for the SYN data. Therefore, we compute the *residual error* for the evaluation on this data as:

8 $$\begin{aligned} e_H = \frac{1}{S_{IW}S_{IH}} \sum _{i = 1}^{S_{IW}S_{IH}}\left\| (\widehat{{\mathbf {H}}}_{k+1}^k)^{-1}\mathbf {x}_i - ({\mathbf {H}}_{k+1}^k)^{-1}\mathbf {x}_i \right\| ^2, \end{aligned}$$

where $\mathbf {x}_i$ is the coordinate of the *i*th position in the image, $\widehat{{\mathbf {H}}}_k^{k+1}$ and ${\mathbf {H}}_k^{k+1}$ are the estimated and GT homographies from F$_k$ to F$_{k+1}$, respectively, and $S_{IW}$ is the width and $S_{IH}$ is the height of a patch.

**Fig. 6:**
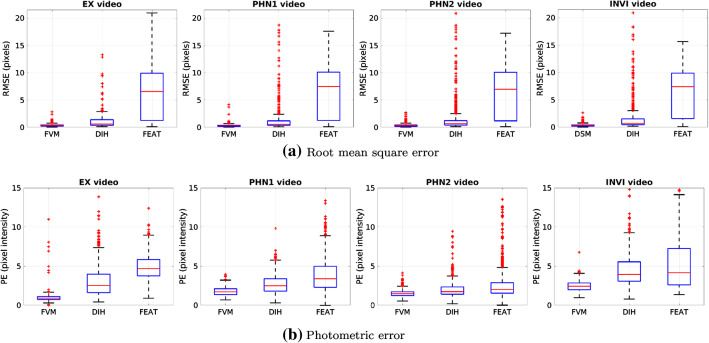
Quantitative comparison of FVM, DIH, and FEAT on the test videos

For the quantitative evaluation of the remaining videos, we report the *root mean square error* between the GT $^{4p}{\mathbf {H}}^A_B$ and estimated $^{4p}\widehat{{\mathbf {H}}}^A_B$ 4-point homographies obtained when the two patches are extracted from a single image (Sect. 4.1). This is given by:

9 $$\begin{aligned} e_R = \left( \frac{\sum _{i=1}^{4}\left[ (\Delta u_i - \Delta {\widehat{u}}_i)^2+(\Delta v_i- \Delta {\widehat{v}}_i)^2 \right] }{4} \right) ^{1/2}, \end{aligned}$$

where $\Delta u_i$ and $\Delta v_i$ are the GT displacements of the four corners and $\Delta {\widehat{u}}_i$ and $\Delta {\widehat{v}}_i$ are the estimated displacements. Finally, we report the photometric error (PE) between patch $P_{k+1}$ and reprojected patch $P'_k$ obtained by warping $P_k$ using the estimated homography $\widehat{{\mathbf {H}}}_{k+1}^k$. The *photometric error* is computed as:

10 $$\begin{aligned} e_P= \frac{1}{S_{IW}S_{IH}} \sum _{i = 1}^{S_{IW}S_{IH}}\left\| P_k' - P_{k+1}\right\| . \end{aligned}$$

We report the box plots for the three error metrics in the next section.

## Results and discussion

Figure 5a–d shows the qualitative comparison results on one circular loop (360 frames) of the SYN sequence. Figure 5e–g shows the quantitative comparison in terms of the residual, root mean square and photometric errors for the complete length of the SYN sequence. We can observe from these visualizations that the drift is minimal in the case of FVM compared to DIH and FEAT. In the case of DIH, tracking is lost in just 30 frames mainly because of random perturbation of the four corners to generate the training data (Sect. 3.2). This results in extremely high mean residual error for DIH (Fig. 5e). Compared to DIH and FEAT, the three error metrics (Fig. 5e–g) are low for FVM which correlates with the observations from the visualizations. For FVM, the median values for residual, root mean square, and photometric errors are 3.88, 0.29, and 2.42, respectively, which are significantly better than FEAT median values (15.67, 7.6, 2.94).

**Fig. 7:**
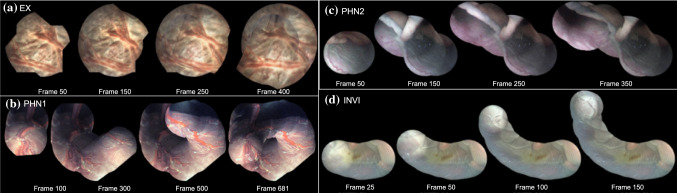
Qualitative results of the proposed FVM

Quantitative comparison of the proposed FVM with FEAT and DIH reporting the root mean square error is presented in Fig. 6a for the EX, PHN1, PHN2, and INVI videos. For the proposed FVM, the median value of the root mean square error for EX is 0.30, PHN1 is 0.27, PHN2 is 0.30, and INVI is 0.29. These values are significantly lower than DIH and FEAT. Error for DIH is also low but not as low as FVM. The root mean square errors for FVM and DIH are particularly low because the optimization of these methods is done on the 4-point homographies, and root mean square error is also calculated using this representation. However, the introduced error in DIH is higher, compared to FVM, mainly because of the random perturbation of the corner points during training. A similar performance trend is observed from the photometric error (Fig. 6b) for which FVM returned the median value of 0.90 in EX, 1.72 in PHN1, 1.46 in PHN2, and 2.41 in INVI videos. Note that the median in the case of FVM for all four test videos is lower than the first quartile (25th percentile) of DIH and FEAT. Moreover, the interquartile range is very small in the case of FVM, depicting that the error at each frame is concentrated in a small range. Compared to FVM, the mean and interquartile range of DIH and FEAT are high because of inaccurate homography estimates resulting in higher reprojection error. These results and observations are in line with the qualitative analysis that is presented in the subsequent paragraphs.

Figure 7 shows the visualization result of the proposed FVM for the EX, PHN1, PHN2, and INVI videos. The visualization results for DIH and FEAT are presented in the supplementary material. EX (unseen data) is of low resolution with blurred frames and captures a spiral scanning motion. FVM created a meaningful mosaic for EX video with minimum drift accumulation over time. PHN1 is the longest video under analysis and has non-planar views without occlusions with the camera following a circular trajectory. FVM managed to generate reliable mosaics with minimum drift which can be observed from frame 681 in Fig. 7b, where loop closure with minimal drift can be seen. Unlike the existing methods that use external sensors for minimizing the drift [26], FVM relies only on image data and generates meaningful mosaics with minimum drift even for non-planar sequences.

PHN2 represents a challenging scenario as it contained highly non-planar frames with heavy occlusions, low resolution, and texture paucity. None of the existing fetoscopic mosaicking literature investigated such a scenario. DIH and FEAT failed to register this sequence (refer to supplementary), while FVM gave promising results. We observe from Frames 250 and 350 (Fig. 7c) that although the generated mosaic can serve well for increasing the FoV, there is a noticeable drift due to heavy occlusions and highly non-planar views. Such errors may be corrected by end-to-end training using the photometric loss [20]. INVI is taken from a TTTS fetoscopic procedure and contains occlusions due to the appearance of the fetus in the FoV, reflection from floating particles, illumination variation, and low resolution. DIH failed to register consecutive frames in this sequence. FEAT lost tracking around 50th frame due to inaccurate feature matches (refer to supplementary). However, FVM (Fig. 7d) managed to generate a meaningful mosaic for the complete duration of the sequence with noticeable drift.

The quantitative and qualitative comparison on five diverse fetoscopic test videos shows that the proposed FVM is capable of handling several visual challenges such as varying illumination, specular highlights/reflections, and low resolution along with non-planar views with occlusions. The proposed FVM solely relied on the image intensity data and generated reliable mosaics with minimum drift even for non-planar test videos.

## Conclusion

We proposed a deep learning-based fetoscopic video mosaicking framework which is shown to handle fetoscopic videos captured in different settings such as simulation, phantoms, ex vivo, and in vivo environments. The proposed method extended an existing image homography method to handle sequential data. This is achieved by introducing a controlled training data generation stage which assumed that there is only a small change in rotation and translation between two consecutive fetoscopic frames. Homography estimates slightly vary between two consecutive frames when selecting patch location randomly during testing due to texture paucity and visual variations; hence, this can introduce drifting error. To handle this issue, we introduced the consistent homography estimation stage that pruned the homography estimate between multiple pair of patches extracted at random from two consecutive frames. Quantitative and qualitative evaluations on five diverse fetoscopic videos showed that, unlike existing methods that are unable to handle visual variations and drift rapidly in just a few frames, our method produced mosaics with minimal drift without the use of any photo-consistency (loop closure) refinement method. Such a method may provide computer-assisted interventional support for TTTS treatment to facilitate the localization of abnormal placental vascular anastomoses by providing an expanded FoV image.

## Electronic supplementary material

Below is the link to the electronic supplementary material.

Supplementary material 1 (pdf 4800 KB)

## Footnotes

1 Surgical Touch Simulator https://www.surgicaltouch.com/.

2 FFmpeg https://ffmpeg.org.
