# Supplementary material for: Deep learning-based fetoscopic mosaicking for field-of-view expansion
Source: Int J Comput Assist Radiol Surg. 2020 Aug 17;15(11):1807–16. doi: 10.1007/s11548-020-02242-8 (PMC7603466; doi:10.1007/s11548-020-02242-8)
Supplement: Supplementary file 1 — Supplementary material 1 (pdf 4800 KB) [file 11548_2020_2242_MOESM1_ESM.pdf]

International Journal of Computer Assisted Radiology and Surgery

Deep Learning-based Fetoscopic Mosaicking for Field-of-View Expansion

Sophia Bano, Francisco Vasconcelos, Marcel Tella Amo, George Dwyer, Caspar Gruijthuijsen, Emmanuel Vander Poorten, Tom Vercauteren, Sebastien Ourselin, Jan Deprest, Danail Stoyanov*

* *Wellcome/EPSRC Centre for Interventional and Surgical Sciences (WEISS) and Department of Computer Science, University College London, London, UK*
Email: sophia.bano@ucl.ac.uk

ELECTRONIC SUPPLEMENTARY MATERIAL

(a) FVM

(b) FEAT

(c) DIH

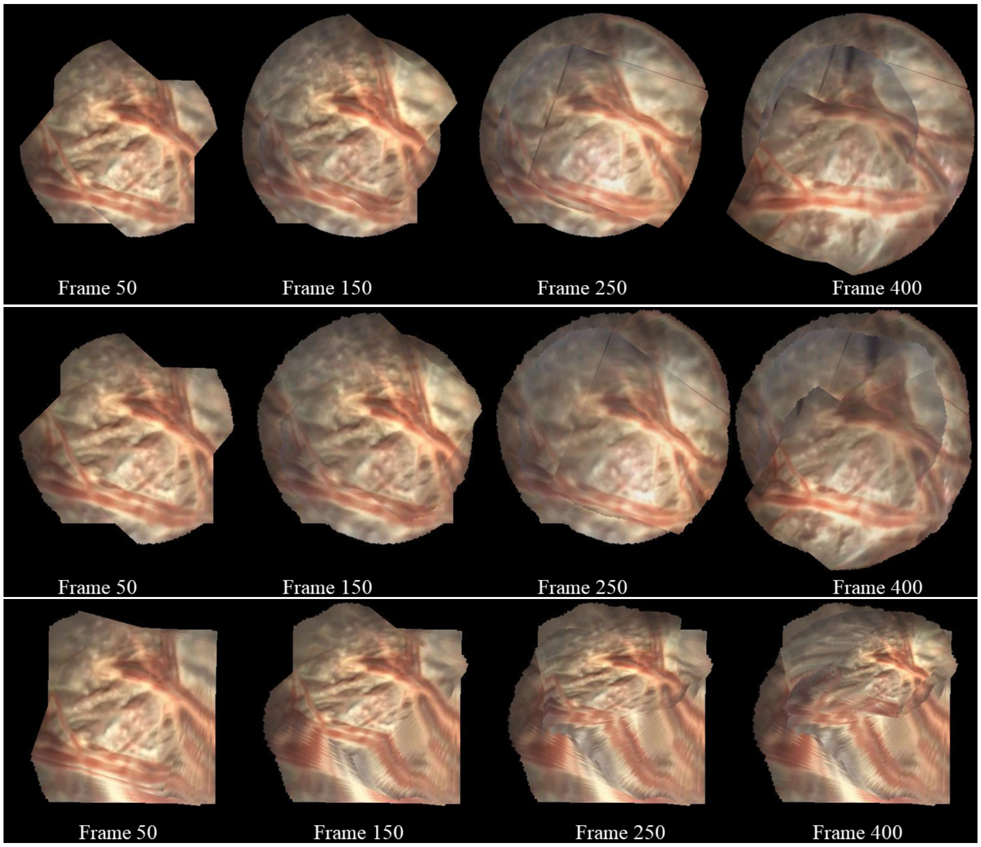

Figure 1. Qualitative comparison of the EX video that contains 404 low-resolution planar frames capturing spiral motion. This is an unseen video as it is not used during training. DIH failed to register in just few frames. FEAT is unable to handle this data and slowly drifts away because of the added challenges due to poor visual quality. Yet the proposed FVM generated a reliable mosaic with minimum drift.

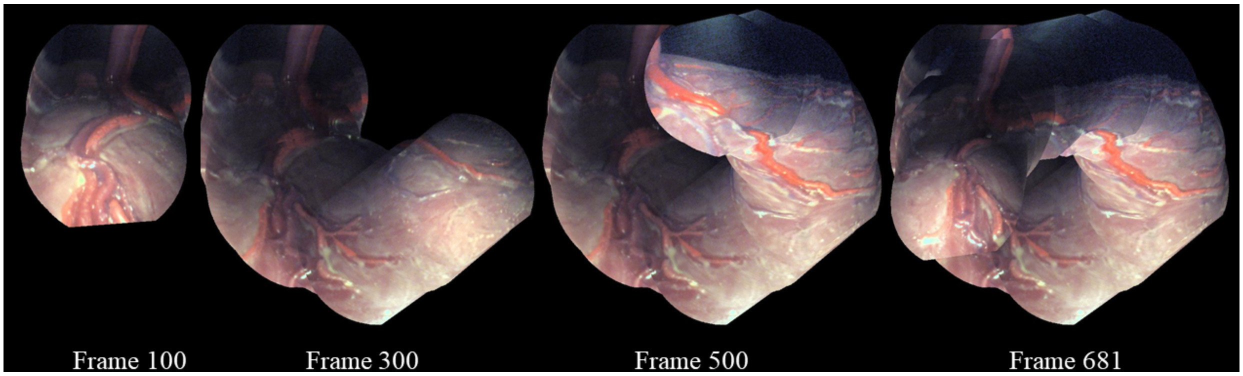

(a) FVM

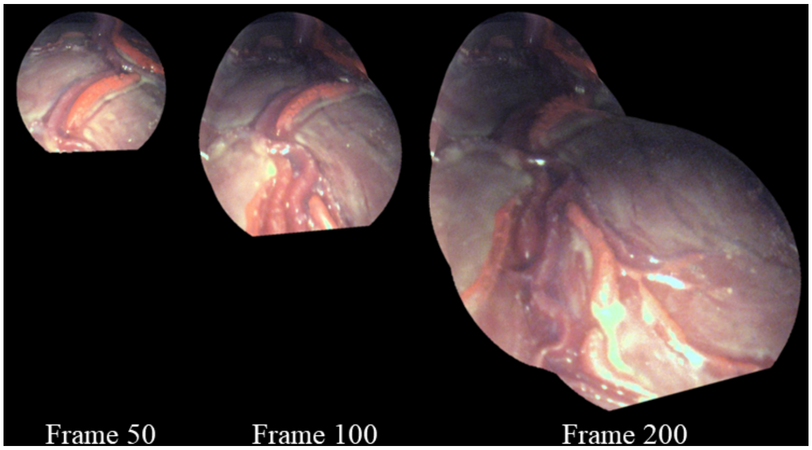

(b) FEAT

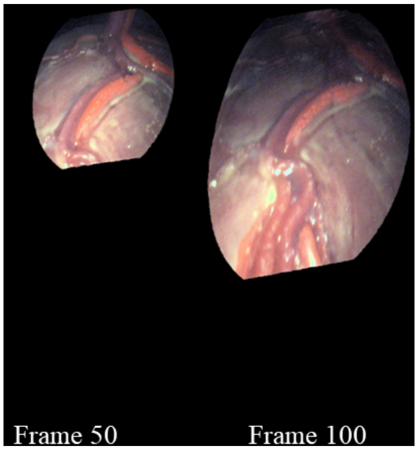

(c) DIH

Figure 2. Qualitative comparison of PHN1 video that contains 681 non-planar frames captured with freehand that followed a circular trajectory. Both DIH and FEAT drifted away after 50 frames due to non-planar views and long-range video. FVM generated mosaic with minimum drift that is verified by observing the loop closure at Frame 681.

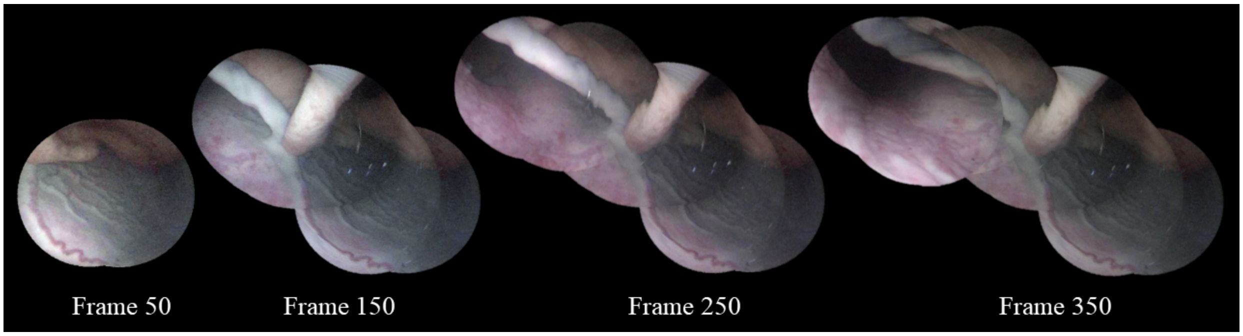

(a) FVM

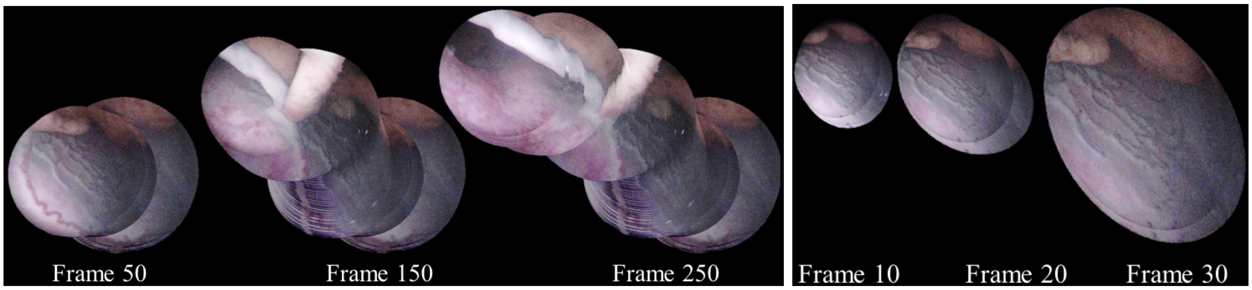

(b) FEAT (c) DIH

Figure 3. Qualitative comparison of PHN2 video containing 350 frames. DIH failed in less than 10 frames. In the case of FEAT, tracking is lost in less than 50 frames as the method did not detect enough reliable features for matching. FVM results are promising compared to FEAT and DIH but introduces drift due to heavy occlusions and non-planar view (contrast enhanced mosaics for better visualisation).

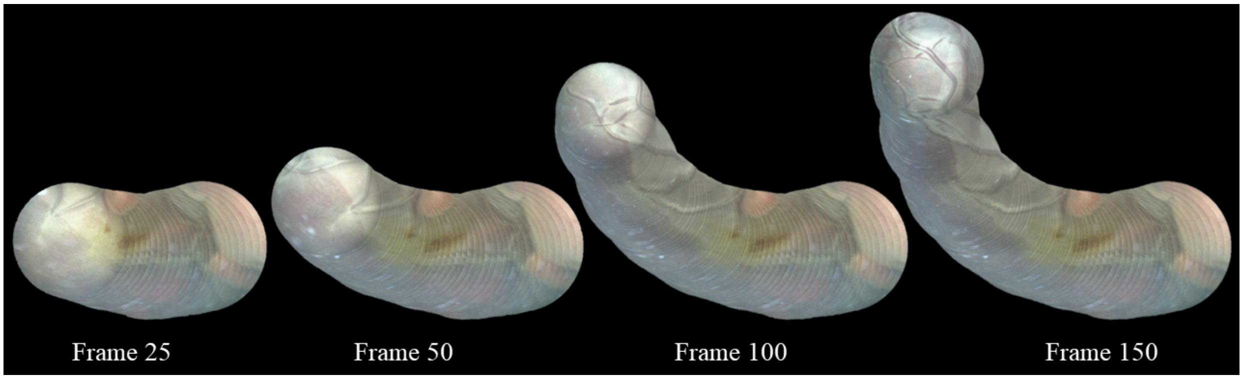

(a) FVM

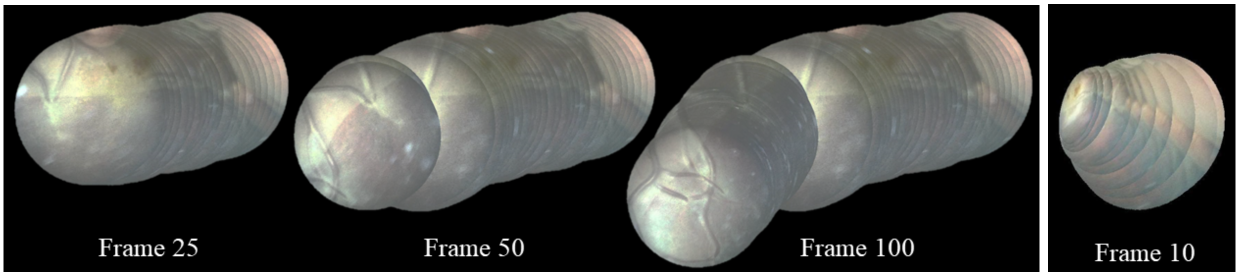

(b) FEAT (c) DIH

Figure 4. Qualitative comparison of INVI video that contains 150 frames having occlusion due to fetus, floating particles, illumination variation and low pixel resolution. DIH failed to register consecutive frames in this sequence. FEAT lost tracking around 50th frame due to inaccurate feature matches. However, FVM gave meaningful mosaic compared to FEAT and DIH.
